# The trigger-information-response model: Exploring health literacy during the first six months following a kidney transplantation

**DOI:** 10.1371/journal.pone.0223533

**Published:** 2019-10-14

**Authors:** Kari Gire Dahl, Eivind Engebretsen, Marit Helen Andersen, Kristin Hjorthaug Urstad, Astrid Klopstad Wahl

**Affiliations:** 1 Department of Interdisciplinary Health Sciences, Institute of Health and Society, Faculty of Medicine, University of Oslo, Oslo, Norway; 2 Department of Transplant Medicine in the Division of Surgery, Inflammatory Medicine and Transplantation, Oslo University Hospital, Oslo, Norway; 3 Department of Quality and Health Technology, Faculty of Health Sciences, University of Stavanger, Stavanger, Norway; Imperial College Healthcare NHS Trust, UNITED KINGDOM

## Abstract

The main objective of this study was to explore how kidney transplant recipients find, understand, and use health information, and make decisions about their health—also known as health literacy. Kidney transplant recipients must take an active part in their health following the transplantation, since a new organ requires new medication and focus on lifestyle to prevent side-effects and signs of organ rejection. Consequently, it is of major clinical relevance to explore how kidney transplant recipients understand and relate to health literacy. Ten kidney transplant recipients were interviewed at three weeks and again at six months post-transplantation. Design and analysis were inspired by constructivist grounded theory. The results of the study are presented through a model consisting of three phases: the trigger phase, the information phase, and the response phase. The participants were influenced by context and personal factors as they moved between three phases, as information seekers, recipients, and sharers. This study illustrates health literacy as an active process. It gives new insight into what motivates kidney recipients to find, share, and receive information, and how a hierarchy of resources is built and used.

## Introduction

Health literacy as a concept has developed over the past three decades, initially focusing on reading and numeracy skills and now covering much broader competencies. The World Health Organization [1] defines health literacy as “the cognitive and social skills which determine the motivation and ability of individuals to gain access to, understand and use information in ways which promote and maintain good health”. They further state that “health literacy implies the achievement of a level of knowledge, personal skills, and confidence to take action to improve personal and community health by changing personal lifestyle and living conditions”. Thus, health literacy brings together different concepts related to what people need in order to make good decisions about their health [2].

Today we know that limited health literacy is associated with numerous negative consequences, both in the general population [3] and also for patients with chronic conditions [2, 4]. Kidney transplant recipients often have a long history of chronic renal disease and comorbidities. Following a transplantation, the recipients depend on lifelong immunosuppressive medication to avoid rejection of the new organ. At the same time, the transplant recipients must be aware of their health in order to monitor signs of rejection and reduce the extent of adverse effects such as infections [5], cardiovascular disease [6], osteoporosis and skin cancer [7, 8]. The available literature on health literacy in kidney transplant recipients is exclusively quantitative, measuring health literacy with generic [9–15] and transplant-specific tools [9, 14, 16]. Studies indicate that kidney transplant recipients constitute a selected group, as the level of health literacy seems to be higher for patients who are awaiting or have already received a kidney transplant compared to other patients with chronic kidney disease [10, 16–19]. However, we do know that limited health literacy in kidney transplant recipients is associated with non-adherence to medication [12, 20], higher creatinine level [14], and comorbidity [18]. Findings also imply an association between low health literacy and lower socioeconomic status [14, 15], lower educational level [13, 14, 19], unemployment, and long-term disability [18].

Existing studies have contributed to our knowledge of health literacy in the context of kidney transplant recipients, yet we call for a deeper understanding of what constitutes health literacy when applied in real-life situations. Further insight into how kidney transplant recipients understand and address health information and make decisions about their own health would have major clinical relevance in patient follow-up. This article takes a bottom-up approach to health literacy. Rather than taking the concept for granted we explore how “health literacy” makes sense from a transplant recipient’s point of view. Through the use of semi-structured interviews and observing interactions with healthcare providers, we focus on the first six months following the transplantation. In this early stage, requirements for adaptation and health literacy skills are challenged, and it is crucial to evolve follow-up programs and initiate interventions of good quality and clinical relevance.

## Methods

### Context

Norway has one nationwide transplantation center where all kidney transplantations are performed and where recipients are followed closely during the first eight postoperative weeks. Recipients remain on the surgical ward for one week before being transferred to the outpatient ward. Patients living close to the hospital can stay at home during this period, while others must stay at the patient hotel. After discharge, a local kidney specialist (nephrologist) follows up with the kidney recipients. All kidney transplant recipients undergo comprehensive, individual patient education starting the first week on the surgical ward, followed by three sessions on the outpatient ward [21].

### Designing the study

In the present study, we chose a qualitative design using semi-structured interviews to explore health literacy in an inductive and situated way. Participatory observations were used to prepare for the first round of interviews. Existing multidimensional definitions of health literacy partially guided the thematic focus concerning both interviews and observations. However, we also sought to move beyond the current, dominant definitions and explore aspects not captured by these. This meant that the participants’ subjective understandings were analyzed as equally plausible and valuable constructions of the world. The study design and analysis were inspired by constructivist grounded theory [22], which follows the inductive, emergent, open-ended, and iterative approach of Glaser and Strauss, but treats data and theorizing as constructed, not discovered [23].

### Patient involvement

A user representative from the National Association for Kidney Patients and Transplant Recipients was involved in the planning and completion of the study. The patient adviser was invited to comment on the interview guide, the analysis of the interviews, and the writing of the article, to ensure that the content was understandable and to discuss whether the findings were recognizable to him as a transplant recipient.

### Sample

Ten kidney transplant recipients were asked to participate in the study by a nurse on the surgical or outpatient ward, approximately 6–10 days post-transplantation. All of the patients accepted the invitation. Since the aim was to capture a wide range of perspectives concerning health literacy, we used purposive sampling to achieve maximum variation (Table 1). The participants came from different areas of Norway and had different socioeconomic and sociodemographic backgrounds as well as different diagnoses. The participants were already part of a larger quantitative study in which they had answered the multidimensional Health Literacy Questionnaire [24] five days post-transplantation. Scores from this instrument were used as selection criteria to invite participants reporting various health literacy challenges and strengths (S1 Table).

**Table 1:** Sample description.

**Age**		28–78 years
**Sex**	Women	5
	Men	5
**Duration of kidney disease**		2–38 years
**Living at home during first interview**		3
**Living at patient hotel during first interview**		7
**Dialysis status pre transplantation**	Pre-emptive dialysis	4
	Peritoneal dialysis	2
	Hemodialysis	4
**Donor status**	Deceased donor	7
	Living donor	3
**Transplantation status**	First time	9
	Second time	1
**Civil status**	Living alone	3
	Living with a partner	7
**Ethnicity**	Norwegian	9
	Non-Norwegian	1
**Level of education**	Completed primary and lower secondary school	1
	Completed upper secondary and/ or vocational school	4
	Less than four years of higher education	4
	More than four years of higher education	1
**Employment status**	Working at time of transplantation	4
	Homemaker	1
	Student	1
	Retired	2
	Disability pension	2
**Diagnosis**	Nephrosclerosis	
	Congenital multiple malformations	
	Secondary amyloidosis	
	Glomerulonephritis	
	Diabetic nephropathy	
	Lupus nephritis	
	Recurrent pyelonephritis	
	Alport syndrome	
	Polycystic kidney disease	

### Data collection

In the process of planning the interviews and the interview guide, we observed the participants in two different consultations with healthcare personnel: one consultation with a nephrologist approximately 7–10 days post-transplantation; and the second at three weeks post-transplantation, in the form of individual patient education with a nurse (Table 2). Communication with healthcare providers is an important aspect of health literacy, and the observations functioned as a relevant basis for asking questions about how the participants experienced interacting with healthcare providers, receiving, evaluating, and asking for relevant information. The interview guide (S2 Table) functioned as a basis for the interview, helping to relate health literacy to specific experiences. However, other reflections and experiences relevant to health literacy were also pursued.

**Table 2:** Overview of data collection.

	Duration	Recordings and notes	Location/ context	Time	Focus
**First observation: generating questions for first interview**	13–35 minutes	Audio recording Field notes and questions for the first interview	One of the first consultations with a nephrologist on the outpatient ward	8–14 days post-transplantation	Observation guide: non-verbal communication, atmosphere and potential questions for interview
**Second observation:** **generating questions for fist interview**	25–45 minutes	Audio recording Field notes and questions for the first interview	The second individual patient education session on the outpatient ward with a nurse	Three weeks post-transplantation	Observation guide: non-verbal communication, atmosphere and potential questions for interview
**First interview**	40–110 minutes	Audio recording Notes about thoughts, non-verbal communication and atmosphere after the interview	1–3 hours after the second observation In a nearby office or in the participant’s hotel room	Three weeks post-transplantation	Interview guide and questions generated in the first and second observations
**Second interview**	75–150 minutes.	Audio recording Notes about thoughts, non-verbal communication and atmosphere after the interview	In the participant’s home or at a place of their choice	Six months post-transplantation	Life-form interview with focus on everyday experiences Interview guide generated by concepts emerging from the first interview

The combination of observation and interview was pilot-tested in a clinical setting before data gathering commenced. KGD undertook the participatory observations and was introduced to the participants as both a researcher and a transplantation specialist nurse. The first round of interviews was conducted three weeks post-transplantation by KGD and MHA; MHA was introduced as a researcher at the transplant clinic. KGD conducted the second round of interviews six months post-transplantation (Table 2). The second round of interviews was inspired by life-form interviews [25], which explicitly focus on experiences in everyday life. The questions were more open-ended than in the first round and were concentrated around how health literacy was applied in real-life situations. The interview guide in this round also contained major themes from the first round, further exploring the concept of triggers, contact, trustworthiness, and continuity, and how this influenced the creation of a possible hierarchy of information resources (S2 Table). Before each interview, the participants were introduced to the term “health literacy” as follows: “health literacy involves how you seek, understand, and use the information that you feel you need to take care of your health; health literacy is about the social support you have around you, how you experience interaction with healthcare personnel and your experiences of navigating the healthcare system; and finally, health literacy is about the different decisions you make that may influence your health”.

### Analysis

The observations were only used to generate questions for the first interviews and were not analyzed further. KGD and an assistant transcribed the interviews. The transcripts were not returned to the participants. KGD undertook the coding, in continuous discussion with the co-authors and with use of NVivo 11. The transcribed material was coded line-by-line, followed by focused coding and theoretical categorizations (Table 3) [22]. Line by line coding involved a close reading of each interview and creating codes that stayed close to the interview data. After this initial phase, a more selective phase began, in which the most significant or frequently occurring codes became focused codes. The focused codes could be short, such as “hierarchy of information resources,” or they could be more elaborating codes (Table 3). The coding phase involved interactive work with constant questioning, commenting, and critical reflections around the analysis by writing memos [22]. Through the coding process and memo-writing, the theoretical categories appeared. The focused codes and theoretical categories from the first interview, such as triggers and the hierarchy of resources, were pursued in the second interview through theoretical sampling [22]. Theoretical sampling was used to elaborate and refine theoretical categories, with the goal of saturating the theoretical categories that appear in the trigger-information-response-model [22]. The theoretical codes and the model were developed through constant comparison within and between codes, categories, memos, and the model. All authors agreed on the analysis and the construction of the trigger-information-response model of health literacy.

**Table 3:** Examples of analysis.

Excerpt from the interview reflecting the theoretical category	Initial coding	Focused coding
**Theoretical category: Person in context**		
*“When you go to the doctor as often as I do now*, *you can wait with the questions for a day*. *But if you’re going to the doctor in a week or two*, *you want to find out everything*. *It’s okay to try to find an answer on the internet*, *but the doctor is best” (9–1)*	Frequency of contact and availability of health care providers influence how she decides about a source of information; less availability increases the chance of using other resources that are lower down in the hierarchy—using the internet instead of the doctor	Context and availability are decisive when seeking information
**Theoretical category: Trigger–phase**		
*“When you read all that*, *everything about those side-effects*, *you feel sick just by reading about it*. *But if a side-effect should occur*, *then maybe…” (6–1)*	He does not seek information that may cause anxiety without it being necessary	The need of a trigger to seek information
*“Every time I meet health care personnel*, *I forget to ask about it [a wound on her breast]*, *because it doesn’t hurt—I can’t feel it”* (8–2).	The absence of pain makes her forget to seek information about the wound on her breast	Absence of pain—the wound does not trigger enough
**Theoretical category: Information–phase**		
*“Someone told us that you can lose the kidney by getting that biopsy* … *But I knew right away that I would ask the doctor*, *‘What are the disadvantages or benefits*?*’ It’s okay to listen to what others say*, *but I don’t believe everything*, *so I checked my information with the doctor” (9–1)*	Information from fellow patients triggers the need to confirm the information using a resource higher up in the hierarchy	Hierarchy of information resources
*“I know them [nurses] very well and call them if I have any questions*. *So that’s where I find or get the information I need*. *It’s mostly the nurses I’ve had contact with*. *When I call them they recognize my voice*, *‘Hey*, *how are things*?*” (2–1)*	He knows the nurses and they know him—this becomes a natural source of information	Continuity involved mutual knowledge—a natural source of information
**Theoretical category: Response–phase**		
*”You’re not as obsessed about it as you were in the beginning perhaps*, *looking for symptoms or thinking*, *have I peed less than normal for the last three hours*, *is there anything wrong now*?*” (3–2)*	He does not look for symptoms—are less sensitive towards situations that may trigger	Sensitivity towards triggers decreased with time and experience
*"I realized that after a transplant it was quite normal to put on some weight*. *You have the risk of getting osteoporosis if you do not walk a little and cycle or exercise a bit (*…*)*, *and diabetes*, *yes*, *so we cut out chocolate and sweets*, *mostly*. *But that’s the reason*, *otherwise I would probably not have lost weight” (10–2)*	The risk of side-effects triggers him to change his diet, start exercising, and lose weight	Information triggered the motivation to change lifestyle

### Ethical considerations

The research project was approved by the Norwegian Regional Committee for Medical and Health Research Ethics (Reference: 2016/1485/REK Sør-Øst C), and by the Data Protection Officer at Oslo University Hospital (Reference: 2016/14592). The Head of the Department of Transplantation Medicine also granted approval.

All participants signed a written informed consent form before participating in the study. The five nurses and six doctors that were observed along with the participants during consultations also signed written informed consent forms.

KGD is a nurse in the transplantation ward but did not have contact with the participants while they were on the ward. Line-by-line coding was employed to avoid having the researcher’s assumptions influence the process of analyzing the material [22].

## Results

**Fig 1:**
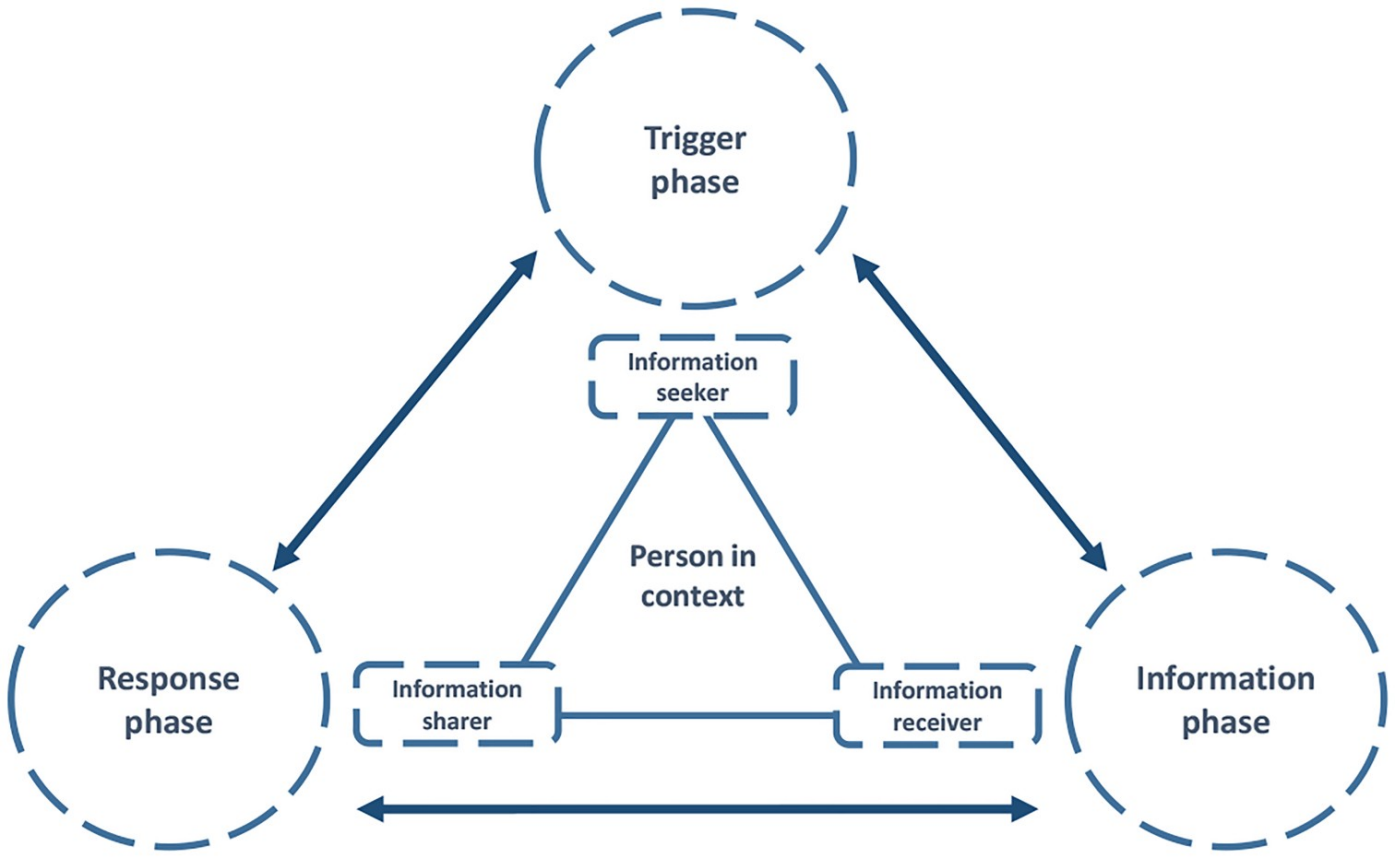
The trigger-information-response model.

The main categories that appeared during the interviews are presented as an empirical model (Fig 1) that consists of three phases: **the trigger phase**, **the information phase,** and **the response phase.** The participants were influenced by context and personal factors as they moved between three phases, as information seekers, recipients, and sharers. This study illustrates health literacy as an active process. There is no linear relationship between the three phases, meaning that the response phase could be an endpoint, or a trigger could result in the participant going back and forth between the information phase and the response phase several times.

### The person in context: Seeking, receiving and sharing information

*The person in context* constitutes the core of the model and is conceptualized as an information seeker, recipient, and sharer concerning health literacy. However, the person in context comprises contextual factors like social support, place, and time since the transplantation. During their stay at the hospital, health issues were often discussed with fellow patients, and the threshold to approach health professionals was low since the participants had planned consultations several times a week. As the participants traveled back home, health issues and decisions about when and with whom to consult were often discussed with a spouse or other family members. Also, a greater distance to health care professionals naturally increased the threshold for making contact. Context also involved personal factors like experience, knowledge, culture, health condition, expectations, and feelings of responsibility and self-confidence.

As *information seekers*, the participants emphasized the importance of balancing information. The following quote exemplifies the experience of several of the participants, as this participant states what information he needs, but also how he limits the amount of information so as not to become overwhelmed.

”I would like to know the creatinine, maybe the urea, but I don’t want a print-out of the blood test results. I prefer to be well when I am well and do other things than go into the disease with things that might trigger anxiety and worries.”

(4-1)

How the participants acted as *information recipients* was influenced by how they preferred to learn, their memory capacity, and the timing of the information. One participant explained how she needed to focus on one thing at a time. It was difficult for her to process information that was more relevant for the future.

“I didn’t read about transplantation beforehand either. I thought everything has its own time. It was the same when I had to learn about PD [peritoneal dialysis]. I saw it, but I couldn’t have done it myself. I distanced myself from it. I need to be there. I need to experience it myself. It was too much to take everything in before I was there.”

(5-1)

The participants were not passive recipients of information, but also *information sharers*. They expressed having considerable knowledge of and experiences with their health conditions and wanted to be heard.

### The trigger phase: The occurrence and interpretation of a health literacy trigger

When exploring what health literacy meant to the participants, triggers appeared as an important concept. A trigger could be an incident or condition that triggered the need to seek and receive information or help, or to share information. As information seekers, the participants considered it important to focus on their normal life, and not on life as a transplant recipient or a patient. Thus, a trigger was an important initiator in the search for information. One participant describes a typical example:

“Over the past five years, I’ve been fine with my kidneys. Suddenly everything changed very quickly, then I started searching for more information.”

(9-1)

Triggers could also occur when receiving information. For example, during patient education, participants learned that fever could be a sign of organ rejection or infection. This information established a new trigger, which subsequently led the participants to always consult the local nephrologist. Information from other patients could also serve as triggers, such as hearing that a fellow patient’s blood test results were better than theirs, thereby creating the need for more information about how to interpret their own results. Symptoms such as pain or fever appeared to be particularly important, both as an information resource and the participants’ experiences of triggers. Obtaining information about a health condition without simultaneous bodily symptoms could make the participants interpret the information as less serious and subsequently less triggering. The following quote exemplifies this:

“My creatinine is a bit high. They [the doctors] think so. I think it is a little odd that I don’t feel it. When you don’t feel anything, that nothing hurts or anything, I think everything is probably okay.”

(1-2)

Triggers were also important when sharing information. One participant had experienced major bleeding as a complication from biopsy and surgery. Her fear of experiencing bleeding again triggered her to always share this information in relevant situations. During the first six months following the transplantation, the participants described a change in their experience of triggers. They described how sensitive they were in the early postoperative phase, where small things became serious triggers. Six months later, with more experience and knowledge, they were less sensitive.

### The information phase: Processing information and creating a hierarchy of resources

The information phase explains how the participants made decisions about internal or external resources that could provide information or help when they experienced a health literacy trigger. How the participants chose a resource for information depended on the context, personal factors and how they interpreted the trigger. A resource was *internal* if a participant’s personal knowledge and experience were sufficient to respond to a trigger. How the participants chose between *external* resources depended on the experiences of contact and trustworthiness. *Contact* was influenced by language, availability, and threshold. *Trustworthiness* was influenced by the participants’ perception of the resource’s competence, the feeling of being taken seriously, and their experience of personal connection and usefulness. Finally, *continuity* in health care influenced both contact and trustworthiness.

The nephrologist was a natural information resource for several of the participants. Other typical resources included the general practitioner and other medical specialists, the nurses at the local hospital, written information from the transplant ward, family members, fellow patients and the internet (mostly Google and Facebook). Different triggers generated different needs and gave rise to changes in the resource hierarchy. Making a hierarchy of information resources was a way for participants to select and arrange information, as this participant explains:

“There is a ton of information. I don’t mind that, but it needs to be sorted a little. If you take in and emphasize equally all the information you get from everywhere I think you’ll be walking in circles. And therefore, I talk to the doctor, I think he knows best.”

(10-1)

To establish a resource, contact had to occur. Speaking the same language was crucial, whether it was the same national language or the doctor translating medical language into one the patient could understand. Availability was a key factor and was experienced very differently from one participant to another. One participants had his nephrologist’s private mobile phone number and had a very low threshold for contacting his doctor. Another participant stated that she preferred to consult her general practitioner because the nephrologist was hard to reach:

“I could call him if there was anything but I don’t call a doctor that I have scarcely met before, I cannot [make myself] do that. I started going to my general practitioner.”

(7-1)

All the participants talked about having a certain threshold for seeking information or help, especially when consulting the doctor—the trigger had to be perceived as strong enough to warrant crossing that threshold. For some, the threshold could be quite high and the feeling of “bothering” healthcare personnel could be uncomfortable due to low self-confidence. If both availability and self-confidence were low, the trigger needed to be very strong for the participant to make contact. As one participant said,

“I think I feel like I don’t want to bother anyone. There are many things I have never asked about. But I know I have poor self-esteem and that it affects me in many areas.”

(8-2)

Trustworthiness depended on the participants’ perception of a resource’s competence and their feeling of personal connection. The feeling that healthcare personnel did what they could—showing commitment to them as patients and taking them seriously—was essential. The resource also had to be perceived as useful to be at the top of the resource hierarchy. Continuity in care was another factor that repeatedly came up in the interviews and seemed crucial when participants were establishing, choosing, and trusting a resource. This was especially true when choosing to consult healthcare personnel, as continuity ensured the security of being known and sharing common knowledge about the participant’s health condition (Table 3).

### The response phase: Processing a response

The response phase describes the responses that occurred after the trigger phase and/or after the information phase and depended on how the trigger was perceived by the participant and what kind of information the participant had been seeking, sharing, and giving. The responses the participants described depended on whether the information was interpreted as adequate or not and could result in a search for more information or help from other resources. Questions such as “How long will my kidney last?” were hard to answer and could result in anxiety. One participant experienced discontinuity and a lack of trusted resources in his post-transplant follow-up. This absence of a hierarchy offering alternative resources made him feel paralyzed.

A typical response was a health-related action. One example is a participant who learned about the adverse effect of immunosuppressive medication during patient education and lost 14 kg during the following six months. Information about the risk of cardiovascular disease and obesity became a trigger that was reinforced by observing fellow patients gaining weight during their eight weeks at the patient hotel.

Readjusting sensitivity towards a trigger was another response, as exemplified by one participant who had experienced recurrent episodes of chest pain. This trigger made him seek help at the hospital several times and each time he received the same answer—that nothing was wrong. Experiencing the same trigger several times and repeatedly searching for information slowly changed his response. His sensitivity concerning the information his body gave him decreased because nothing happened, and he developed an explanation as to why the pain occurred and acquired an acceptance of it.

“I think it’s because of all the surgery I had as a kid, I have scars and stuff here [pointing at scars on his chest]. Now I can ignore it completely if I feel any stinging. I’ve had it for such a long time now without anything bad happening.”

(3-2)

An important part of the response was the growing knowledge and experience that became especially evident during the second round of interviews. The knowledge and experience were situated and meaningful for the individual participants and involved a selective process and an interpretation of information based on context and personal factors. One example is a participant who had a prior history of substance abuse. She found that the taking of blood samples from her arm triggered her desire for drugs. She shared this information with a trusted healthcare provider, and together they found a solution that worked, taking blood samples from her foot instead. This knowledge was unique and very important for that participant in her context:

“Blood samples were taken from my arm and it triggered the whole thing, I just dived into it. This happened every single time, it became so demanding and tiring. But suddenly one day I thought, ‘Oh my God, I have feet.’ Then I tried my feet. Nothing. I did not notice anything afterward. Now I use my feet every time.”

(8-2)

Six months of experience and gathering information had developed the participants’ individual knowledge and experience of being kidney transplant recipients, and they knew more about what symptoms were side effects of medications, and what could be signs of organ rejection. Their evolving knowledge made them less sensitive to situations that would have created triggers in the early phase. They also felt more secure about when and where to find information and help.

## Discussion

In this Norwegian study, we aimed to elucidate what health literacy may comprise in the context of kidney transplantation, using a qualitative design. The main findings are presented as a model that may offer a supplement to our understanding of health literacy as a process moving between and across a trigger phase, an information phase, and a response phase. During this process, context and personal factors influenced all the three phases: what constituted triggers, how a hierarchy of resources changed and was utilized, and how the participants in the study responded and made decisions about their health. The model also emphasizes the person in context as an information seeker, receiver and sharer.

We found that triggers worked as important facilitators for the participants to start the process of seeking information or help. Jordan et al. [26] also suggest that a “trigger” or a “health event” is needed for people to be motivated to seek out or be receptive to health information. Research on information-seeking behavior has found that individuals must recognize a gap in their knowledge—often signaled by a feeling of anxiety or a need to act—before they are motivated to search for information [27]. Furthermore, Jordan et al. [26] found that prior health experiences and knowledge affect when and where individuals seek information. This supports our findings that experience and knowledge influenced the participants’ experience of triggers, and where they went to find information or help. We also found context and other personal factors such as culture, expectations, health condition, feelings of responsibility, and self-confidence to be important inhibitors or facilitators in all three phases of health literacy. For instance, low self-confidence and the desire to avoid being a burden for healthcare providers could prevent some of the participants from addressing their needs, or lead them to seek other sources of information than healthcare professionals. Leung et al. [28] found the same in patients with diabetes; the concern that they might be wasting professionals’ time made patients hesitate to indicate their need for health information. If the threshold for making contact is high, it will not only hinder patients from obtaining good-quality information or help, it may also prevent them from acquiring knowledge and create a barrier for good communication with healthcare providers. This may further reduce the opportunity to take an active part in treatment decisions.

In the creation of a resource hierarchy, we found contact, trustworthiness, and continuity of care to be decisive factors that could explain why the participants chose one resource above another. However, we could not rank these factors in order of importance, apart from ascertaining that different triggers required different resources and that this would probably also influence which of the three were most important for the participant. Earlier studies have found that individuals do not necessarily consult the resource that they trust the most, but rather turn to the available ones. For example, people choose the internet due to availability, even when they trust their doctor more [29, 30]. In our study, fever seemed to be the ultimate trigger, always resulting in the participants calling the local nephrologist. Participants were taught repeatedly during patient education that fever should be interpreted as a serious trigger, and our findings emphasize how information may create triggers and motivate patients to establish a resource for help or information.

As information seekers, receivers and sharers, findings indicate that the participants were selective. Selection occurred when participants chose one information resource over another, creating a hierarchy of resources to which they turned in different situations. Selection was also important, as health information was translated into contextual and personal knowledge that was meaningful for the participants. Part of the selection process was also to avoid information that might cause anxiety and stress. The literature on health information-seeking has long been concerned with why people avoid information [31], and both seeking and avoiding information may be motivated by anxiety reduction [32]. An important discussion relates to whether avoiding information may be a sign of having adequate or limited health literacy. In Nutbeam’s three categories of health literacy, the most advanced—“critical literacy”—involves critically analyzing information to exert greater control in life events and situations [33]. More information may result in the feeling of losing control, especially if it triggers anxiety. At the same time, this anxiety might be exactly the trigger needed to find more information. The participants were occupied with balancing the information to avoid becoming too involved in potential health issues, and to instead focus on life as “normal”, healthy individuals.

Health literacy reflects the ability to gain access to information and help, but this also depends on the health care offered and the possibility of establishing a hierarchy of quality resources. Paasche-Orlow and Wolf [34] argue that the healthcare system might be too complex and difficult for patients to navigate. It may also lack continuity or trustworthy resources, causing people to turn to lesser-quality resources or not search for information at all. The participants in this study emphasized the importance of continuity, contact, and trustworthiness when choosing one resource over another. This is of clinical relevance, as healthcare personnel may focus on how to facilitate continuity, contact, and trustworthiness, and establish a low threshold for making contact. By targeting at these factors, healthcare personnel might reduce existing differences in the utilization of healthcare services, especially when these differences are caused by personal factors such as low levels of self-confidence or a lack of knowledge.

By moving back and forth between the three phases, existing personal experience and knowledge were confronted with new experience and knowledge. In this way, the participants evolved an individual knowledge and experience that was meaningful in specific contexts. Lonergan [35] uses the verb «knowing» instead of the noun “knowledge”, and suggests that knowledge is not something you discover but an activity—“something that you *do”* (p.529). This may be transferred to the contextual knowledge and experience that the participants developed through the active process of moving between the phases. Knowledge is found to be an essential part of health literacy but is usually described more generally as a set of skills [36], such as having a certain vocabulary for and conceptual knowledge about how the body works [37]. This kind of knowledge is more visible and easier to measure and influence with interventions. The more situated “knowing” is nevertheless an important part of health literacy, and exploring this knowing might give us a better understanding of the complexity and factors that influence health literacy.

Our empirical model focuses on when, why, and how the kidney transplant recipient decide to seek, receive or share information. These results contribute important knowledge to clinical practice. A deeper understanding of triggers as important initiators in health literacy, and the mechanisms behind choosing a resource for help or information, might be transferable to other kidney transplant recipients and give us a broader understanding of what *motivates* in the process of searching for, receiving, and sharing information.

### Strengths and weaknesses of the study

Few studies explore health literacy qualitatively through patient experiences, and currently, this study seems to be one of the few to explore health literacy in the context of kidney transplant recipients. As such, this study helps fill an important knowledge gap. There is an increasing number of kidney transplant recipients of non-Norwegian ethnicity, and existing research indicates that ethnic subgroups might experience several challenges related to finding, understanding, and using health information. Language and cultural barriers may also hinder good communication with healthcare providers. This study only includes one participant of non-Norwegian ethnicity and does not provide comprehensive knowledge about how this affects health literacy in the context of kidney transplant recipients. Hence, additional studies are needed to explore health literacy in different ethnic subgroups.

## Supporting information

S1 Table Participants answers to the Health Literacy Questionnaire. (DOCX)

S2 Table Interview guide 1 and 2. (DOCX)
