# Supplementary material for: The trigger-information-response model: Exploring health literacy during the first six months following a kidney transplantation
Source: PLoS One. 2019 Oct 14;14(10):e0223533. doi: 10.1371/journal.pone.0223533 (PMC6791550; doi:10.1371/journal.pone.0223533)
Supplement: S1 Table — (DOCX) [file pone.0223533.s001.docx]

**S1 Table:** Participants answers to the Health Literacy Questionnaire

|  |  | Participants (1-10) answers to the Health Literacy Questionnaire | | | | | | | | | |
| --- | --- | --- | --- | --- | --- | --- | --- | --- | --- | --- | --- |
| Health literacy domains | **Range** | **1** | **2** | **3** | **4** | **5** | **6** | **7** | **8** | **9** | **10** |
| 1. Feeling understood and supported by healthcare providers  *Having at least one health care provider that you trust, and knows you well, and that can assist in decision making.* | 1-4 | 3 | 3,5 | 4 | 4 | 3 | 3 | 2,5 | 3 | 3 | 3,75 |
| 2. Having sufficient information to manage my health  *Have enough good information to look after my health.* | 1-4 | 4 | 3 | 3,25 | 3,5 | 3 | 3 | 2 | 2,75 | 2,75 | 3 |
| 3. Actively managing my health  *Spending time on actively managing health. Making plans and actively engage in healthy activities*. | 1-4 | 4 | 2,8 | 2,4 | 3,8 | 3 | 3 | 3,4 | 2,8 | 2 | 4 |
| 4. Social support for health  *Feel understood and supported in relation to health. Have access to people that can help if necessary.* | 1-4 | 4 | 3,4 | 3,6 | 3,8 | 3,2 | 3,2 | 1,8 | 2,8 | 3 | 3 |
| 5. Appraisal of health information  *Check the quality of information and compare different sources.* | 1-4 | 3,2 | 2,6 | 2,6 | 3,8 | 2,6 | 3 | 1,8 | 2 | 2,2 | 2 |
| 6. Ability to actively engage with healthcare providers  *Healthcare providers understand my problems and I am able to have meaningful and good discussions with healthcare providers.* | 1-5 | 5 | 4,4 | 4,4 | 4 | 4,6 | 4,2 | 2,8 | 4 | 3,6 | 4,8 |
| 7. Navigating the healthcare system  *Find the healthcare you need, what the best care is and what you are entitled to* | 1-5 | 4,5 | 3,83 | 3,5 | 4,17 | 3,33 | 4 | 2,5 | 3,83 | 3 | 4,83 |
| 8. Ability to find good health information  *Find up to date and understandable information from different sources.* | 1-5 | 3,6 | 3,6 | 3,2 | 4,2 | 3,2 | 4 | 2,8 | 3,6 | 3 | 4,4 |
| 9. Understand health information well enough to know what to do  *Correctly filling out forms, understand and follow instructions from healthcare professionals, read and understand medical labels and other health information* | 1-5 | 3,8 | 4 | 4 | 4,2 | 4,2 | 4 | 3,2 | 3,8 | 3,2 | 4,6 |

Each participant had answered the Health Literacy Questionnaire (HLQ)[1]. The questionnaire contain 44 questions across 9 independent domains where each domain contains 4 to 6 statements [1]. In the first five domains, respondents select one of four responses to a statement: ‘strongly disagree’, ‘disagree’, ‘agree’, and ‘strongly agree’. Within the last four domains, the participants selected one of five responses: ‘cannot do or always difficult’ ‘usually difficult’, ‘sometimes difficult’, ‘usually easy’, and ‘very easy’. The questionnaire gives a mean domain-specific score, calculated by adding each of the answers in one domain and divide the scores by the number of items in the specific domain. A higher score indicates higher HL.

# References:

1. Osborne RH, Batterham RW, Elsworth GR, Hawkins M, Buchbinder R. The grounded psychometric development and initial validation of the Health Literacy Questionnaire (HLQ). BMC public health. 2013;13(1):1.
