# Supplementary material for: The trigger-information-response model: Exploring health literacy during the first six months following a kidney transplantation
Source: PLoS One. 2019 Oct 14;14(10):e0223533. doi: 10.1371/journal.pone.0223533 (PMC6791550; doi:10.1371/journal.pone.0223533)
Supplement: S2 Table — (DOCX) [file pone.0223533.s002.docx]

**S2 Table:** Interview guide 1 and 2

| Interview guide 1  The observations were used to tailor the questions and to generate additional questions relevant to the research question. The interview guide was used as a base to open up for other experiences related to health literacy. Examples of follow-up questions in parenthesis.   - Can you describe how you have experienced the consultations and teaching situations with the health care providers so far?   [From the observation: How did the patient behave? Did he/she look strained/comfortable?] (Does the participant have any other experiences with receiving health information or patient education?).   - Was it easy to understand what the nurse or doctor meant/ was there anything that was hard to understand?   [From the observation: Did the doctor/nurse use difficult words? Did the participants have any questions?]  (Do the participant have relevant experiences from other situations?).   - What did you get out of the consultations/ patient education?   [From the observation: did the participant seem interested during the consultation? What information was meaningful to the participant?]   - Did you feel motivated for patient education? - (What motivates you to receive information?). - What is important to you when you receive information or patient education? - Have you had any questions during any of your consultations that you did not ask?   [From the observation: did the participants have any questions?]   - Are there any topics that are difficult to talk about or to ask about? - Are there any thing in your daily life that make it difficult to concentrate on the information that you get from health providers?   (Have the participant experienced something that can make it had to concentrate, for example an organ rejection or maybe something in his/her private life?).   - If you have any questions on the way home today, or on the way back to the hotel, what will you do to answer those questions?   (Has the participant been in a situation with need for help or information?)   - How are your family or friends concerned with information regarding life after a kidney transplant? - How significant was the meeting with other kidney transplant recipients, with regard to acquiring knowledge about having a kidney transplant? - [+ Other questions generated by the observation situation, like questions they had or stories they told.]   Interview guide 2   - How have you been since the last time we spoke? (Explore the ‘trigger’ concept and the hierarchy of information resources)   - Have you experienced any situations where you needed information, knowledge or help? Can you tell me about that situation?   - Have you experienced any situations regarding your health where you knew exactly what to do? Can you tell me about that situation?   - Have you been in any situations where you were unsure what to do or where to seek information? Can you tell me about that situation? - How did you experience your first consultation at your local hospital after you got home?   - (Explore the feeling of contact, trustworthiness and continuity: Do they have any preferences when they search for information or help?) - How was your last consultation at your local hospital?   - (Explore the feeling of contact, trustworthiness and continuity: Do they have any preferences when they search for information or help?) - Do you have any contact with other kidney transplant recipients? - Social relations:   - How does others influence the decisions that you make regarding your own health?   (Explore context)   - Do you use different sources of information if you seek to answer a question?   - (Explore the hierarchy of resources and different assessments regarding various information sources) |
| --- |

Supplementary table1: In interview 1, examples of possible questions from the observations are written in brackets [ ]. In interview 2, themes that we pursued from interview 1 are written in parenthesis.
